# Methods of optical spectroscopy in detection of virus in infected samples: A review

**DOI:** 10.1016/j.heliyon.2022.e10472

**Published:** 2022-08-29

**Authors:** Muhammad Izzuddin Rumaling, Fuei Pien Chee, Abdullah Bade, Nur Hasshima Hasbi, Sylvia Daim, Floressy Juhim, Mivolil Duinong, Rosfayanti Rasmidi

**Affiliations:** a Faculty of Science and Natural Resources, Universiti Malaysia Sabah, Jalan UMS, 88400 Kota Kinabalu, Sabah, Malaysia; b Faculty of Medicine and Health Science, Universiti Malaysia Sabah, Jalan UMS, 88400 Kota Kinabalu, Sabah, Malaysia; c Faculty of Applied Sciences, Universiti Teknologi MARA Sabah Branch, Kota Kinabalu Campus, 88997 Kota Kinabalu, Sabah, Malaysia

**Keywords:** Virus detection, Optical spectroscopy, Ultraviolet (UV) spectroscopy, Infrared (IR) spectroscopy, Raman spectroscopy, Fluorescence spectroscopy

## Abstract

Due to the recent COVID-19 pandemic that occurred worldwide since 2020, scientists and researchers have been studying methods to detect the presence of the virus causing COVID-19 disease, namely SARS-CoV-2. Optical spectroscopy is a method that employs the interaction of light in detecting virus on samples. It is a promising method that might help in detecting the presence of SARS-CoV-2 in samples. Four optical spectroscopy methods are discussed in this paper: ultraviolet (UV), infrared (IR), Raman spectroscopy and fluorescence spectroscopy. UV and IR spectroscopy differ in wavelength range (less than 400 nm for UV, more than 700 nm for IR). Raman spectroscopy involves shift in wavelength due to scattering of light. Fluorescence spectroscopy involves difference in wavelength between absorbed and emitted light due to vibrational relaxation. These four methods had been proven to differentiate healthy samples from virus-infected samples. UV spectroscopy is useful in determining presence of virus based on 260 nm/280 nm absorbance ratio. However, its usefulness is limited due to its destructive properties on virus at sufficiently high intensity. Meanwhile, IR spectroscopy has becoming popular in studies involving virus samples. Mid-infrared (MIR) spectroscopy is most commonly used among IR spectroscopy as it usually provides useful information directly from spectral data. Near infrared (NIR) spectroscopy is also used in studying virus samples, but additional methods such as principal component analysis (PCA) and partial least squares (PLS) are required to process raw spectral data and to identify molecules based on spectral peaks. On the other hand, Raman spectroscopy is useful because spectral data can be analyzed directly in identifying vibrational modes of specific molecules in virus samples. Fluorescence spectroscopy relies on interaction between viral particles and fluorescent tags for the detection of virus based on improvement or quenching of fluorescent signal. Due to non-invasive properties of virus samples, IR, Raman and fluorescence spectroscopy will be used more often in future studies involving virus detection in infected samples.

## 1 Introduction

Coronavirus (CoV) is a virus characterized by spike-like projections on its surface that can be viewed under an electron microscope to give a crown-like appearance (Singhal, 2020). CoVs have long genomes of up to 32 kilobases, and expansion of genomes is believed to be partially regulated by an increase in replication fidelity (Forni et al., 2017). All CoVs share a similar genome organization, with two large overlapping reading frames: ORF1a/b and ORFS. ORF1a/b encodes 16 nonstructural proteins, while ORFS has four major structural proteins: spike (S), envelope (E), membrane (M) and nucleocapsid (N) (Zhou et al., 2020). Spike protein in CoVs is vital in receptor binding and determination of host tropism and transmission capacity. It is functionally divided into two domains, S1 and S2, both responsible for receptor binding and fusion of cell membrane respectively (Lu et al., 2020).

CoVs are divided into four genera, according to genome characteristics: α-CoV, β-CoV, γ-CoV and δ-CoV (Ciotti et al., 2020; Zhu et al., 2020). Seven human coronaviruses (HCoV) were identified and fall within α-CoV and β-CoV. Two α-CoV (HCoV-NL63 and HCoV-229E) and two β-CoV (HCoV-OC43 and HCoV-HKU1) usually trigger common cold, but they may also cause severe lower respiratory tract infections, especially among children and elderly (Ciotti et al., 2020; De Sabato et al., 2019). Severe acute respiratory syndrome coronavirus (SARS-CoV) and Middle East respiratory syndrome coronavirus (MERS-CoV) are HCoV categorized under β-CoV. SARS-CoV is an aetiological agent that triggered severe respiratory disease and has caused an outbreak in China in 2002 (Su et al., 2016). Meanwhile, MERS-CoV has caused another outbreak of severe respiratory disease centred at Middle East in 2012 (Raj et al., 2014). A novel coronavirus, namely severe acute respiratory syndrome coronavirus-2 (SARS-CoV-2) was identified in 2019 and has caused an ongoing pandemic worldwide. SARS-CoV-2 isolated from lower respiratory tract samples of a patient was revealed to be β-CoV according to deep sequencing (Zhou et al., 2020; Zhu et al., 2020).

Coronavirus disease-19 (COVID-19) is a contagious respiratory disease caused by severe acute respiratory syndrome coronavirus-2 (SARS-CoV-2). The disease emerged in Wuhan, China at the end of 2019, before affecting at least 100 countries globally (Haynes et al., 2020). The transmission rate can be quantified using basic reproduction number (R-naught, or R_o_), adapted from mathematical models. A study showed that the average R_o_ value of COVID-19 is 3.28 (Liu et al., 2020). Although R_o_ value is expected to fall within a range similar to SARS due to similarities in pathogen and region of exposure, several studies have reported different R_o_ values for COVID-19, often much higher than SARS or MERS (Cao et al., 2020; Liu et al., 2020; Shen et al., 2020; Tang et al., 2020; Viceconte and Petrosillo, 2020). Three commonly accepted modes of COVID-19 transmission are via small airborne microdroplets, larger respiratory droplets, and direct contact with contaminated surfaces (Morawska et al., 2020). Transmission mode via small airborne microdroplets is more contagious as small droplets carrying viral content are more affected by air current than gravitation, effectively transmitting droplets up to tens of meters away (Morawska and Cao, 2020). Transmission of COVID-19 becomes more likely in the case of confined space and poor quarantine environment. A study revealed that SARS-CoV-2 has a general secondary attack rate of 16.3%, which is higher than other coronavirus species such as SARS-CoV and MERS-CoV (Li et al., 2020).

The reported symptoms of COVID-19 varies from mild to severe, with some cases resulting in death (Dzieciatkowski et al., 2020). A study showed that the most common symptoms of COVID-19 include fever, cough, fatigue, shortness of breath, sore throat, sputum and myalgia (Guan et al., 2020). COVID-19 becomes more severe in patients with chronic diseases such as heart disease, diabetes, cancer, obesity, chronic kidney disease and chronic obstructive pulmonary disease (Hacker et al., 2021). However, there have been cases where infected individuals do not experience any of these symptoms, which are well known as asymptomatic carriers. These individuals can spread COVID-19 towards highly vulnerable individuals such as elderly, young children, pregnant ladies, smokers and immunocompromised patients (Elengoe, 2020). Because of this, social distancing has become one of the solutions preferred by government in mitigating transmission of COVID-19. One study predicted that social distancing prevents hospitals' overflow by reducing the number of patients requiring ICU care per day, implying that social distancing reduces the transmission rate of COVID-19 (Greenstone and Nigam, 2020). Other methods include travel ban and restrictions, temporary closure of frequently visited places, avoiding 3Cs (Crowded places, Confined spaces, Close conversation) and practising 3Ws (Wash hands, Wear masks, Warn against risks) (Ganasegeran et al., 2020).

Detecting SARS-CoV-2 in patients helps isolate only infected patients and avoid false positives, resulting in unnecessary treatment of uninfected individuals. Polymerase chain reaction (PCR) is the most commonly used method in detecting SARS-CoV-2 in samples (Orooji et al., 2021). PCR has the ability to determine the presence of SARS-CoV-2 with high accuracy. However, obtaining the results usually requires as long as 48 h and multiple steps with varying temperature and chemicals. Rapid Test Kit Antigen (RTK-Ag) is another commonly used method in detecting presence of SARS-CoV-2 based on specific protein virus or antigen. A study by Kalil et al. (2021) showed that RTK-Ag has high specificity. However, this method is prone to false negative, as it was observed that the negative predicted value (NPV), or probability of correctly identifying negative sample, is only 66.7% (Kalil et al., 2021).

Optical spectroscopy is a non-invasive method that employs interaction of light on samples. Spectroscopy is sometimes accompanied by pattern recognition model to improve performance in virus detection. Based on previous research studies, spectroscopy has shown promising results in rapidly detecting presence of virus with high accuracy (Bilal et al., 2017; Fernandes et al., 2018; Khan et al., 2018; Roy et al., 2019; Sakudo et al., 2012). Research involving spectroscopy in detecting SARS-CoV-2 is of the utmost importance with the demand for rapid and robust detection. This paper presents review mainly on methods of virus detection in infected samples by means of spectroscopy. This paper will highlight four spectroscopy methods: ultraviolet (UV), infrared (IR), Raman and fluorescence spectroscopy. These methods will be discussed in terms of theory, techniques in past studies and evidence on the techniques.

## 2 Ultraviolet (UV) spectroscopy

### 2.1 Theory of UV spectroscopy

Ultraviolet (UV) is a type of radiation with wavelength ranging between 10 nm and 400 nm (Chen and Xu, 2021). It consists of radiation having higher energy compared to visible light. UV spectrum is divided based on two standards. The most commonly used standard involves dividing UV spectrum into UVA (320–400 nm), UVB (280–320 nm) and UVC (200–280 nm) (Surjadinata et al., 2017). Major portions of UVA and small portions of UVB enter Earth’s atmosphere via sunlight. This is because Earth’s atmosphere absorbs most UVB radiation in sunlight and the ozone layer in the stratosphere absorbs all UVC radiation (Seyer and Sanlidag, 2020). UV spectrum can also be divided according to another standard: near UV (NUV) (300–400 nm), middle UV (200–300 nm) and far UV (122–200 nm) (Chen and Xu, 2021).

### 2.2 Past research applying UV spectroscopy techniques

UV spectroscopy involves the absorption of visible light and ultraviolet radiation associated with the excitation of electrons from lower to higher energy levels, in both atoms and molecules (Atole and Rajput, 2018). In a study by Porterfield and Zlotnick (2010), nucleic acid and protein spectra can be differentiated quantitatively using 260 nm/280 nm absorbance ratio. Nucleic acid tends to have an absorption peak of around 260 nm, which leads to higher 260 nm/280 nm absorbance ratio. In contrast, protein maximally absorbs UV radiation with a wavelength close to 280 nm, which leads to lower 260 nm/280 nm absorbance ratio. This guideline has been used in past studies in determining the type of viral particles in samples (Minamikawa et al., 2021; Piva et al., 2020; Porterfield and Zlotnick, 2010). For example, a study by Minamikawa et al. (2021) used SARS-CoV-2 sample contained in several different media such as Eagle’s minimal essential medium (EMEM), fetal bovine serum (FBS) and phosphate buffer saline (PBS). It was shown that all media except PBS without FBS exhibit absorbance peak of 280 nm, suggesting that UV absorption of virus is dominated by protein. Furthermore, it was also shown that viruses inside EMEM (10% FBS) exhibit a higher absorption peak at 280 nm than viruses in other media. In another study by Piva et al. (2020), a sample containing pure hRSV M2-1 protein is verified by ensuring lower 260 nm/280 nm absorbance ratio.

Measurement of UV absorbance spectra in a sample is primarily affected by Rayleigh scattering. This occurs because the scattering of light in spherical solutes is proportional to $\lambda^{-4}$ (Porterfield and Zlotnick, 2010; Rüdt et al., 2019). Due to Rayleigh scattering, UV with shorter wavelength is much more prone to be scattered. Furthermore, Rayleigh scattering becomes more apparent when the diameter of spherical solute is smaller. In a study by Porterfield and Zlotnick (2010), the uncorrected readings of 260 nm/280 nm absorbance ratio in protein vary from 0.59 to 0.71. By applying correction for Rayleigh scattering, the reading for 260 nm/280 nm absorbance ratio is consistent at about 0.6, which is similar to findings from previous studies (Glasel, 1995; Goldfarb et al., 1951).

**Figure 1:**
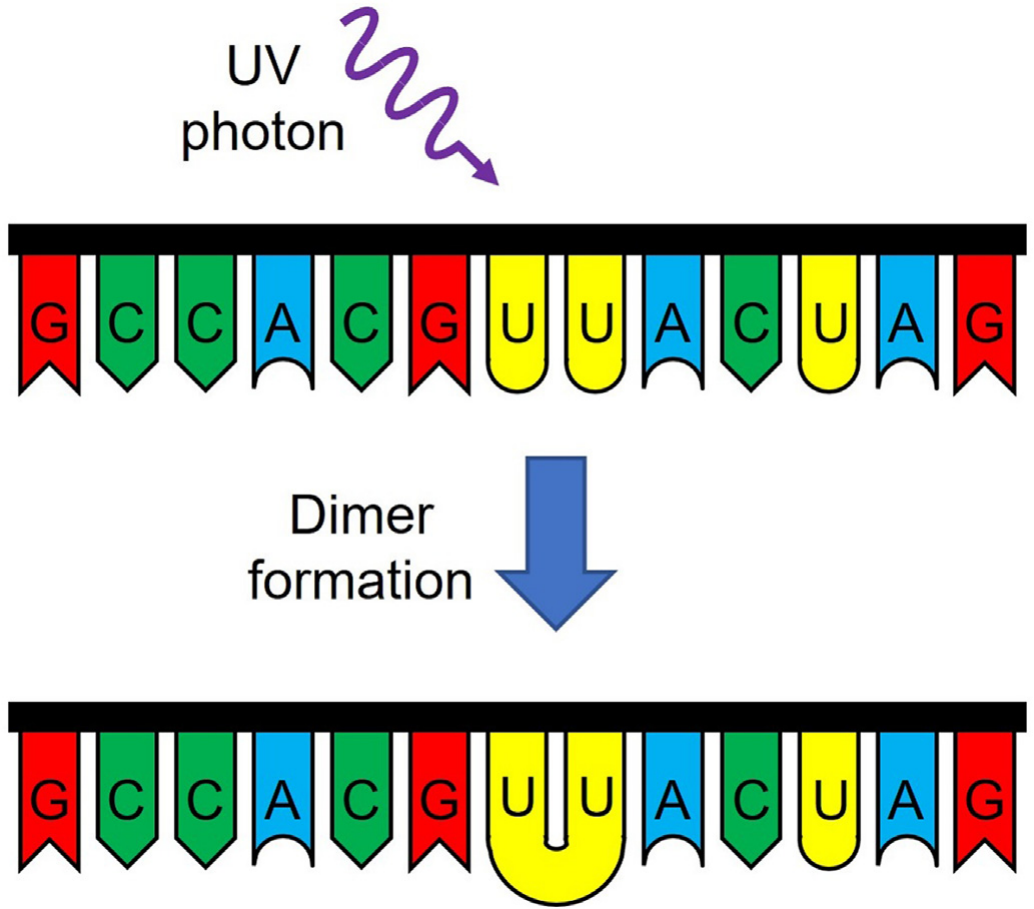
Formation of pyrimidine dimers in RNA.

UV absorbance spectra is not extensively studied for viral samples, especially in recent studies. Depending on the intensity, UV may have destructive effects on the virus. UV spectrum is generally used as disinfectant because it has well-known antiviral effect (Heßling et al., 2020). The disinfectant mechanism involves the use absorption of UV radiation by RNA which forms pyrimidine dimers, as depicted in Figure 1 (Budowsky et al., 1981; Heßling et al., 2020). The most commonly used light source for disinfection is the mercury-vapor lamp, which emits UV radiation at 254 nm. This peak falls under UVC and is very close to maximum absorption peak of RNA at 260 nm (Heßling et al., 2020). Another study by Minamikawa et al. (2021) further explained that UV radiation at 265 nm and 280 nm requires less radiation dose to inactivate the virus than UV radiation at 300 nm. This is also because RNA and protein in a virus absorb more radiation at absorbance peak of 260 nm and 280 nm respectively (Heßling et al., 2020; Porterfield and Zlotnick, 2010).

## 3 Infrared (IR) spectroscopy

### 3.1 Theory of IR spectroscopy

**Figure 2:**
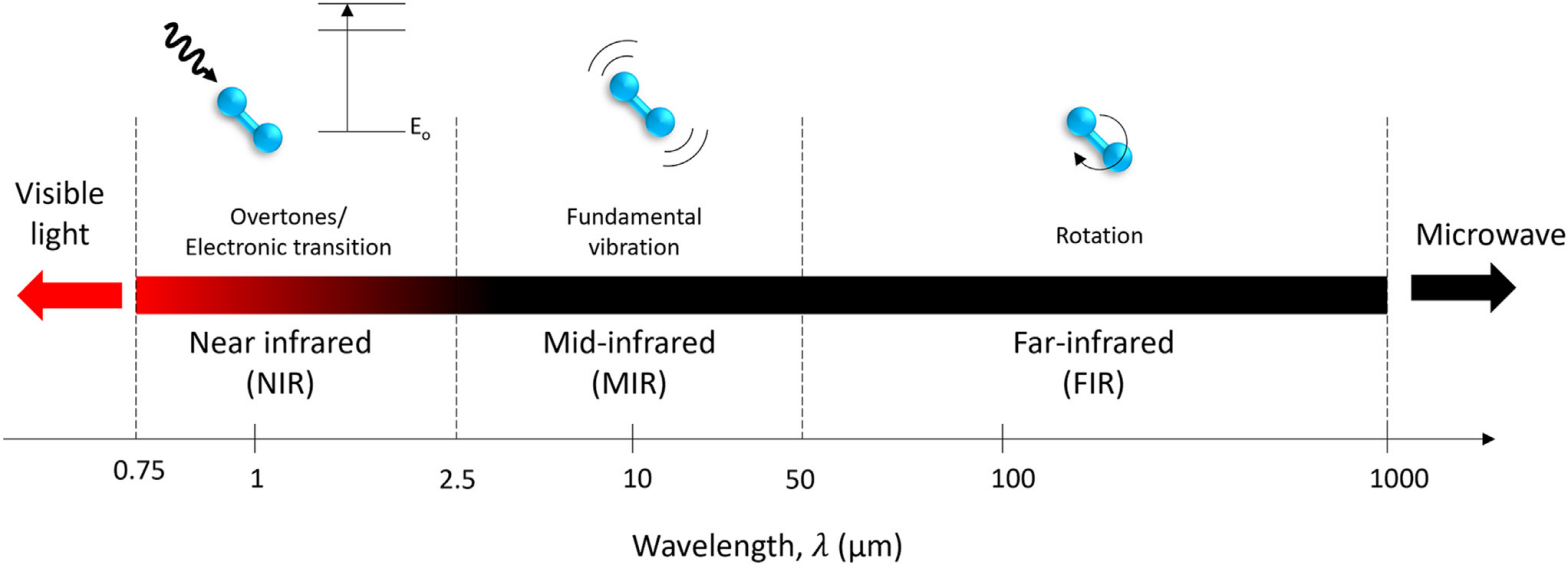
Range of infrared radiation and application in studying molecules.

Infrared (IR) is a type of radiation with wavelength above 750 nm. IR typically has lower photon energy compared to visible light. IR is mainly divided into three types: near infrared (NIR), mid-infrared (MIR) and far infrared (FIR). Radiation is categorized as NIR if its wavelength ranges between 750 nm and 2500 nm (wavenumber from 13333 cm^−1^ to 4000 cm^−1^) (Pasquini, 2018). NIR consists of high-energetic photons invisible to the naked eye, but is energetic enough to cause an electronic transition in molecules (Ozaki, 2012; Pasquini, 2018). NIR is sensitive to overtones and a combination of vibrations in a molecule (Hasbi et al., 2022; Veerasingam et al., 2021). Meanwhile, MIR consists of radiation whose wavelength ranges from 2500 nm to 50000 nm (wavenumber of 4000 cm^−1^ to 200 cm^−1^) (Barth, 2007). MIR is used to study the fundamental vibrations of a molecule (Veerasingam et al., 2021). In addition, FIR consists of radiation with a wavelength between 50 μm and 1000 μm (wavenumber of 200 cm^−1^ and 4 cm^−1^) and is also known as terahertz frequency regime (Barth, 2007). It is used to study the rotation of a molecule (Mukherjee and Gowen, 2015; Veerasingam et al., 2021). Figure 2 illustrates the infrared spectrum's infrared range and its application in studying the molecules.

IR spectroscopy involves using radiation in the infrared region to study the composition of samples. NIR and MIR are commonly used in recent studies involving IR spectroscopy of the virus. NIR and MIR are more commonly used compared to UV as they are non-invasive to virus samples. However, MIR is more commonly used in spectroscopic studies than NIR because it can recognize the change in dipole moment of a molecule (Wiercigroch et al., 2017). Sometimes, MIR is accompanied by Fourier transform (FT) to convert detection signal into actual spectra, hence, the name Fourier transform infrared (FTIR) emerged.

### 3.2 The past studies applying IR spectroscopy technique

Table 1 shows previous studies employing NIR and MIR spectroscopy in virus samples. Certain studies employed additional methods to make the observed peaks in spectra clearer.

**Table 1:** List of previous studies employing NIR and MIR on virus samples.

Author	Virus samples	Type of infrared	Wavelength (nm)/Wavenumber (cm^−1^)	Additional methods	Main findings
Erukhimovitch et al. (2011)	HSV-1, HSV-2 and VZV	MIR (FTIR Absorbance)	4000–800 cm^−1^	Principal component analysis (PCA)	• During the early stages of development, infected samples exhibit higher spectral intensity in the 1220-1260 cm^−1^ region. The intensity at this region significantly decreased at the late stage of development. • Samples during the early stages of development have little to no peak at 1023 cm^−1^ (attributed to carbohydrates), but this peak significantly increased during late stages of development.
Sakudo et al. (2012)	Influenza virus	NIR (Absorbance)	600–1100 nm	Principal component analysis (PCA)	• The sharp peaks of 850, 950 and 1030 nm, as well as broad peaks between 660-740 nm are essential in discriminating influenza-infected sample from healthy samples.
Fernandes et al. (2018)	Zika	NIR (Absorbance)	350–2500 nm	Partial least square (PLS) model	• There is a noticeable difference in absorbance spectra between healthy and Zika-infected samples. • Eight important wavelengths (1000, 1413, 1515, 1711, 1801, 1893, 2109, 2246 nm) are observed in PLS regression coefficient plot. • Peaks at 1000 and 1801 nm may either be due to Zika virus or error in spectrometer used. • Peaks at 1413 and 1515 nm is associated with O–H and N–H overtones, which might correspond to glycoproteins in viral envelope.
Roy et al. (2019)	Hepatitis B and C	MIR (Attenuated total reflectance - FTIR)	600–4200 cm^−1^	Second derivative normalized spectra plot	• Based on second derivative normalized spectra plot, the dominant bands that differentiate healthy samples from HBV-infected samples are 1646, 1631 and 985 cm^−1^. • The dominant bands that differentiate healthy samples from HCV-infected samples are 1646, 1631, 1078 and 989 cm^−1^.
Dou et al. (2020)	MS2/HSV-2	MIR (Absorption via AFM-IR)	1640–1660 cm^−1^ 1022–1087 cm^−1^	-	• Vibrational bands of 1660 cm^−1^ and 1517 cm^−1^ are caused by proteins found in HSV-1. • Vibrational bands of 1020–1057 cm^−1^ and 1258 cm^−1^ are caused by nucleic acids contained in HSV-1. • MS2 exhibits more intense vibrational bands (compared to HSV-1) at 808 and 860 cm^−1^, 1258 cm^−1^ and 1450 cm^−1^, all of which are caused by nucleic acids.
Barauna et al. (2021)	SARS-CoV-2	MIR (FTIR - Absorbance)	650–4000 cm^−1^	-	• An increase in absorbance of infected sample at 1429 cm^−1^ is associated with the virus itself. • Decrease in absorbance of infected samples at 1220, 1084, 1069 and 1041 cm^−1^ is associated with host organism response against infection.
Wood et al. (2021)	SARS-CoV-2	MIR (FTIR - Absorbance)	800–4000 cm^−1^	Second derivative transformed spectra plot, principal component analysis (PCA)	• Positive samples exhibit more intense peaks at 3288, 1658 and 1549 cm^−1^, indicating high protein levels due to viral proteins or antibody • More intense peaks at 1078 and 1249 cm^−1^ are observed in positive samples, both are associated with nucleic acid coming from viral RNA.

### 3.3 Evidence of IR techniques

**Figure 3:**
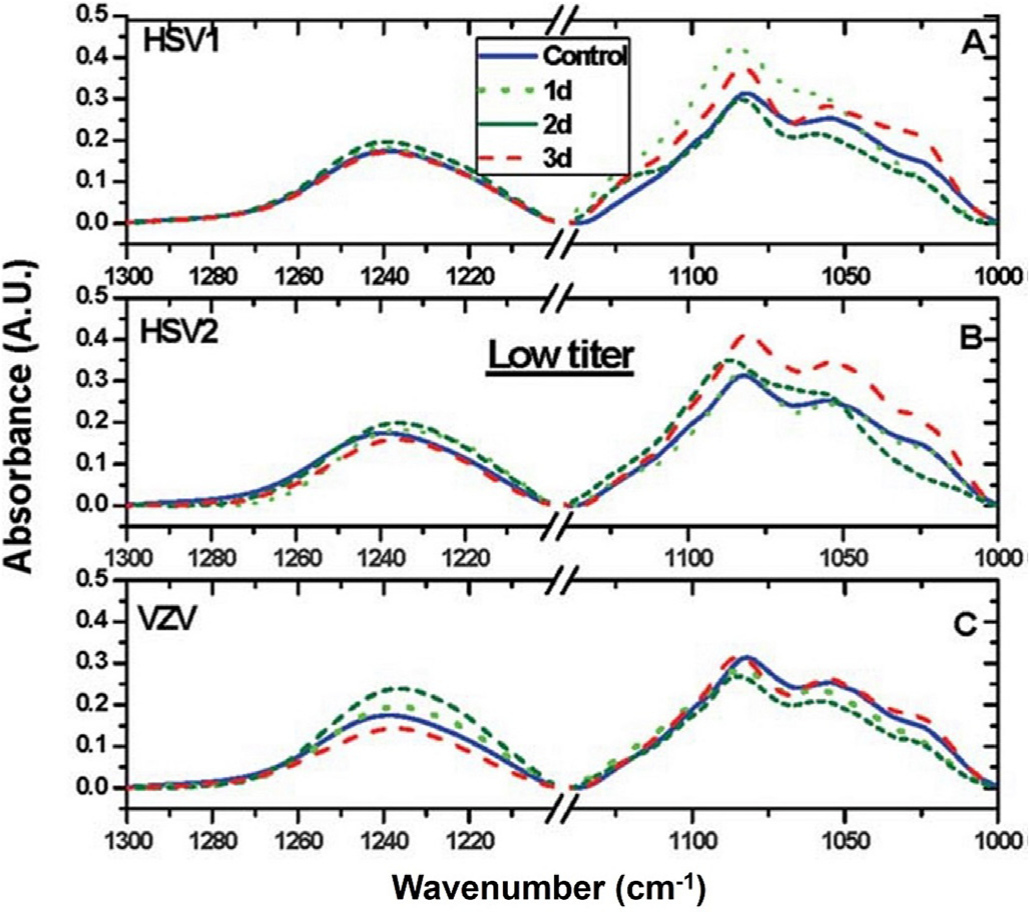
MIR absorbance spectra of control (healthy) sample and infected samples at different stages (Erukhimovitch et al., 2011).

A study by Erukhimovitch et al. (2011) measured FTIR absorbance spectra to differentiate infected samples at different stages of herpes virus development from healthy samples. Regions at 1000–1140 cm^−1^ and 1200–1300 cm^−1^ were focused due to significant changes of infected cells at different stages of development. Based on Figure 3, the absorbance peak at 1240 cm^−1^, representing the specific vibrational mode of phosphate, significantly increases at 1–2 days after infection of the viruses. The specific vibrational mode of phosphate at this peak is related to the nucleic acid (Wong et al., 1993). The increase of absorption peak at 1240 cm^−1^ is most probably caused by the accumulation of free viral nucleic acid at the host cell's nucleus (Erukhimovitch et al., 2011). However, the decrease in peak is observed at 3 days after infection. This is the result of apoptosis and death of cells, where all viral mRNA and most of DNA from host cell degrade (Holman et al., 2000). Furthermore, the absorption peak of 1023.3 cm^−1^ from Figure 3 (attributed to carbohydrates) is not visible during early stage of development compared to an uninfected cell. The disappearance of this peak results from the cell undergoing apoptosis during the early stage of development (Chalmers and Griffiths, 2002; Holman et al., 2000). This peak reappears during the late stage of development most probably due to severe possible damage of cell membrane which highly exposes cytoplasmic components to IR radiation (Erukhimovitch et al., 2011).

**Figure 4:**
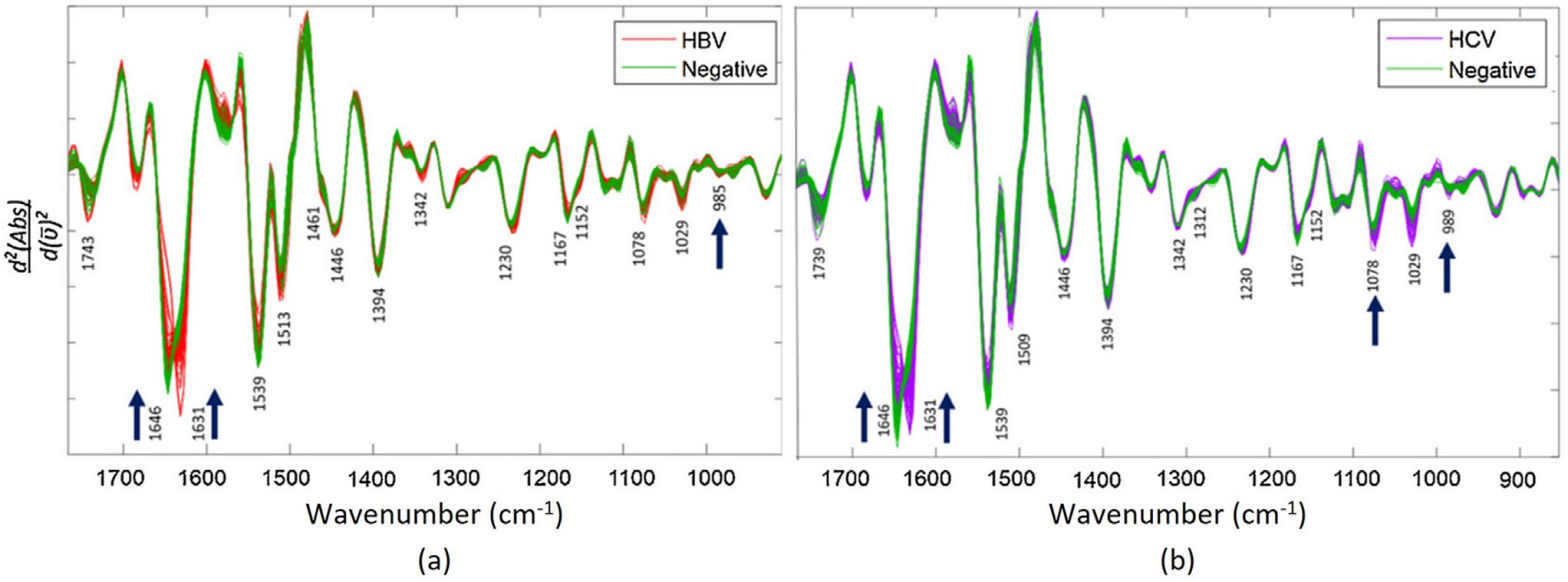
Second derivative normalized spectra plot of (a) HBV-infected sample and healthy sample, and (b) HCV-infected sample and healthy sample (Roy et al., 2019).

Another study conducted by Roy et al. (2019) used attenuated total reflectance FTIR (ATR-FTIR) to diagnose samples infected with hepatitis B virus (HBV) and hepatitis C virus (HCV). The light source used in the study is in MIR region, with wavenumber ranging from 600 cm^−1^ to 4200 cm^−1^. Based on the raw spectra obtained, the second derivative normalized spectra for HBV and HCV are plotted, as in Figure 4. Out of several peaks in second derivative normalized spectra, three peaks (1646, 1631 and 985 cm^−1^) differentiate healthy samples from HBV-infected samples. Meanwhile, four peaks (1646, 1631, 1078, 989 cm^−1^) differentiate healthy samples from HCV-infected samples. These peaks are marked with arrows in Figures 4(a) and 4(b).

The minima band at 1646 cm^−1^ is present in healthy samples while the minima band at 1631 cm^−1^ is present in both infected samples. The more intense minima band of 1631 cm^−1^ in infected samples is the characteristics of a β-pleated sheet which is assigned to inflammation marker immunoglobulin (IgG) (Spalding et al., 2018). It is found that IgG level increases when the patient is infected with either Hepatitis-B or Hepatitis-C disease (Roy et al., 2019). Furthermore, the band of 991 cm^−1^ is observed to be red-shifted in HBV (985 cm^−1^) and HCV (989 cm^−1^) infected samples. This band is also associated with IgG (Petibois et al., 2001), but this band is more intense in HCV-infected samples compared to other samples. In addition, the band of 1078 cm^−1^ may be caused by symmetric phosphodiester stretch of viral nucleic acids, but the spectrum of 1074 cm^−1^ also appears quite strongly in IgG sample (Roy et al., 2019).

**Figure 5:**
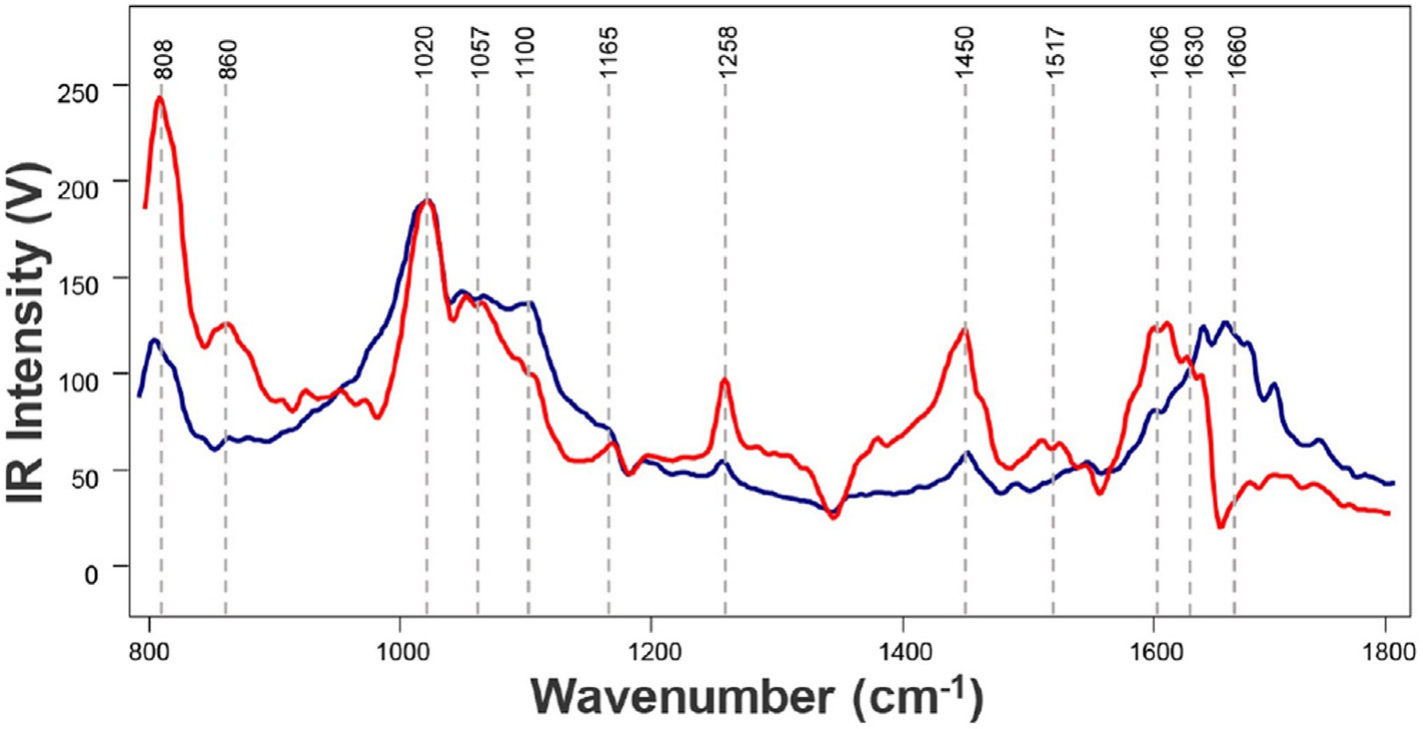
Absorbance of MIR in MS2 (red) and HSV-1 (blue) samples as implied by IR intensity of cantilever oscillation (Dou et al., 2020).

In a study conducted by Dou et al. (2020), MIR absorbance spectra and imaging of MS2 bacteriophage and Herpes simplex virus type 1 (HSV-1) samples are obtained using atomic force microscopy infrared (AFM-IR). Although the authors did not explicitly stated that MIR absorbance is obtained, AFM-IR works by measuring MIR absorbance of the sample based on cantilever vibration in contact with the sample (Dazzi and Prater, 2017). Figure 5 illustrates the absorbance spectra in MIR region for MS2 and HSV-1 samples. HSV-1 contains proteins whose vibrational bands can be assigned to 1660 cm^−1^ (amide I) and 1517 cm^−1^ (amide II). HSV-1 also contains DNA whose nucleic acid exhibits vibrational bands at 1020–1057 cm^−1^ (C–O stretching of deoxyribose residue) and 1258 cm^−1^ (P–O vibration) (Wood, 2016; Zucchiatti et al., 2016). On the other hand, MS2 exhibits very different spectra as observed in Figure 5. Its nucleic acid causes vibrational bands that are more intense (compared to HSV-1) at 808 and 860 cm^−1^ (nucleic acid sugars), 1258 cm^−1^ (P–O vibration) and 1450 cm^−1^ (CH_2_ vibration).

**Figure 6:**
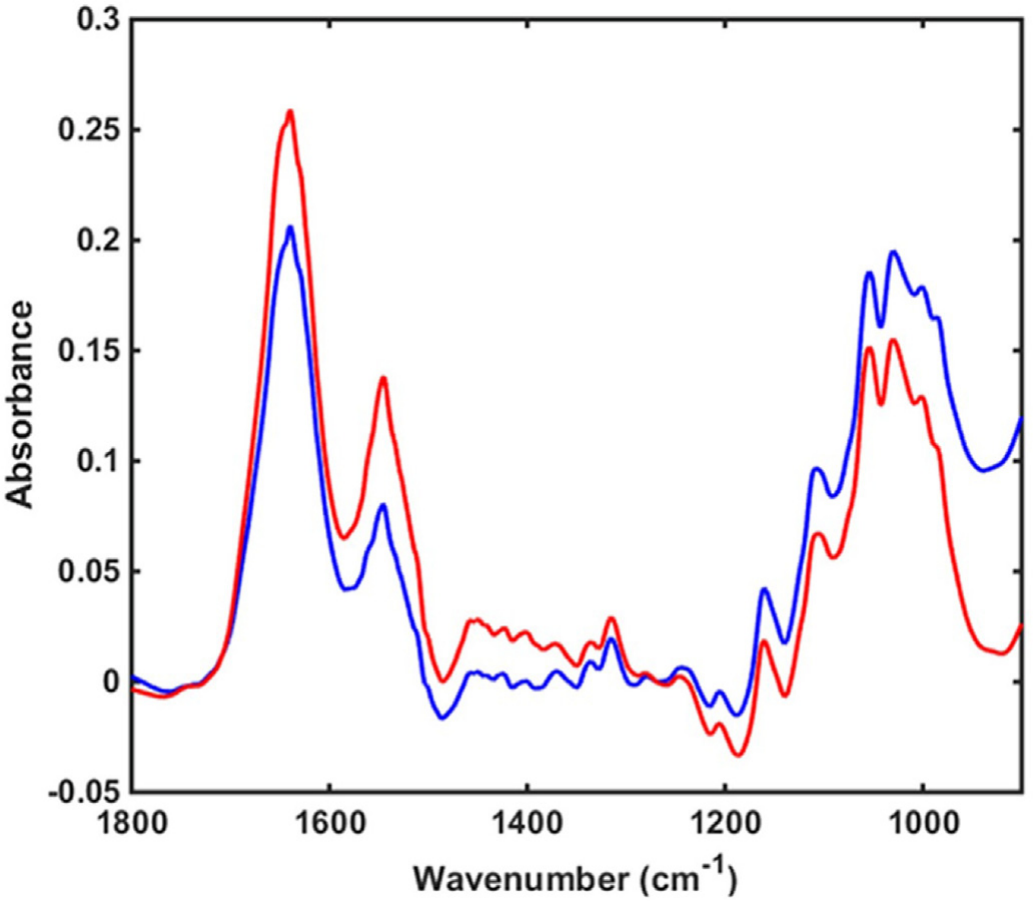
MIR absorbance spectra of saliva on swab (blue) and saliva with SARS-CoV-2 virus on swab (red) (Barauna et al., 2021).

A study conducted by Barauna et al. (2021) used FTIR spectroscopy on SARS-CoV-2 samples with MIR of wavenumber ranging from 4000 cm^−1^ to 650 cm^−1^. The difference between absorbance spectra of saliva samples (with and without SARS-CoV-2) are primarily associated with the nucleic acid. Based on Figure 6, higher absorbance peak of 1429 cm^−1^ in the infected sample is probably due to the virus itself. Meanwhile, the decrease in absorbance peaks located at 1220, 1084, 1069 and 1041 cm^−1^ is probably associated with the response of the host organism against the virus (Barauna et al., 2021).

**Figure 7:**
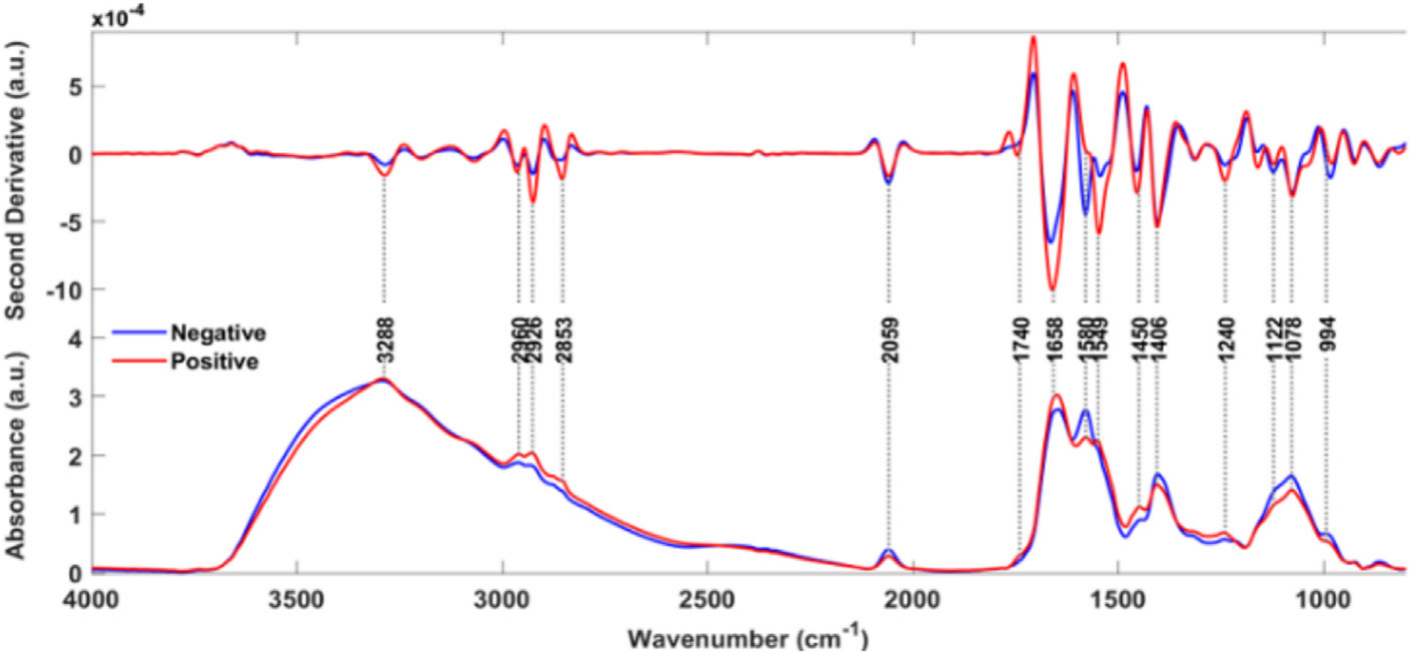
FTIR absorbance spectra and corresponding second derivative transformed plot for positive and negative samples (Wood et al., 2021).

Wood et al. (2021) utilised FTIR spectroscopy to differentiate salivary samples from healthy patients from those infected with SARS-CoV-2. Absorbance spectra and corresponding second derivative transformed spectra for both positive and negative samples are revealed in Figure 7. Strong bands from proteins at peaks of 3288, 1658 and 1549 cm^−1^ appeared more intense in positive samples. It must be noted that saliva has low levels of proteins compared to other biofluids (Schultz et al., 1996). Thus, the significant difference in peaks associated with protein is most likely due to viral proteins or antibodies. Furthermore, positive samples exhibit more intense peaks at 1240 and 1078 cm^−1^, which are associated with nucleic acids (Wood, 2016). These indicate that viral RNA in positive samples contributes to these peaks.

NIR study on virus mostly involves absorption of NIR radiation by virus samples. It is expected that the absorption peaks at NIR region are due to either electronic transition or overtones of certain molecules. However, previous studies involving NIR absorbance found that the difference in NIR absorbance spectra in healthy and infected samples requires additional methods to signify the difference in spectra between both samples (Fernandes et al., 2018; Sakudo et al., 2012). Differences in spectra as observed from processed data can be used to determine the overtones or vibration of specific samples.

In a study conducted by Sakudo et al. (2012), Vis-NIR absorbance spectra are measured on healthy and influenza-infected samples. The spectra are further processed using principal component analysis (PCA) and soft independent modelling of class analogy (SIMCA) to identify predominant absorbance peaks. Sharp peaks (850, 950 and 1030 nm) and broad peaks (660–740 nm) in SIMCA model are predominant to differentiate healthy samples from infected samples. The broad peak between 660 and 740 nm are related to alcohol and C–H aromatic groups (Workman and Weyer, 2007). The alcohol and C–H aromatic group also causes sharp peaks observed at 1030 nm and 850 nm respectively (Williams and Norris, 2001). Meanwhile, another sharp peak at 950 nm is close to the water band (Osborne, 2000). The difference in peaks as expressed by SIMCA model may be caused by various changes such as modulation of protein expression and movement of surrounding cells, which may be caused by the influenza virus and may be similar to effects caused by a respiratory syncytial virus (RSV) (Welliver, 2003). The influenza virus induces cytokines and chemokines, which cause activation of lymphocytes and subsequently causes these changes (Eccles, 2005).

**Figure 8:**
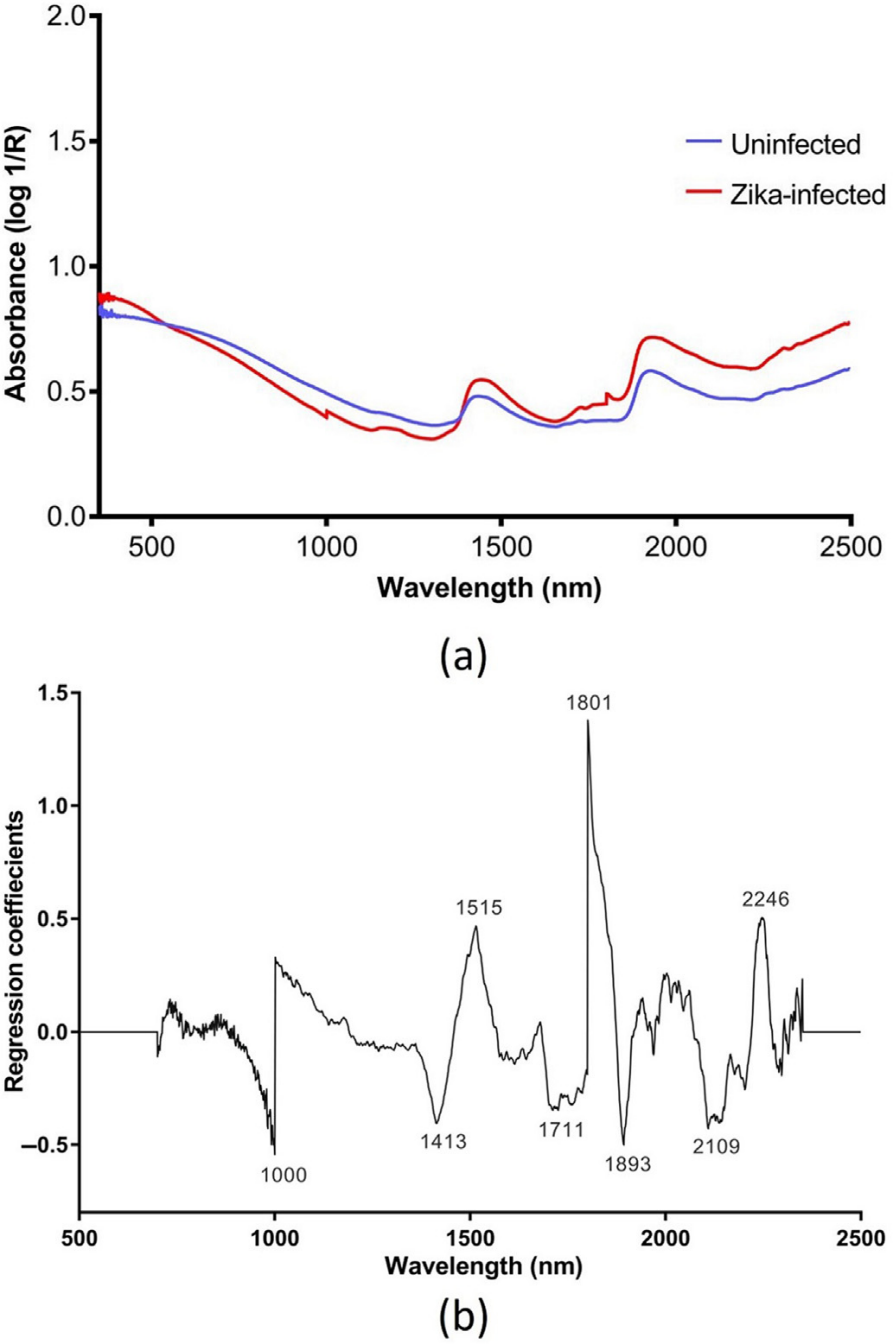
(a) Raw Vis-NIR absorbance spectra of uninfected and Zika-infected samples, and (b) regression coefficient of PLS model based on NIR absorbance spectra from 700 nm to 2350 nm (Fernandes et al., 2018).

Another study by Fernandes et al. (2018) studied on Vis-NIR absorbance spectra of Zika-infected *A. aegypti* mosquitoes. Figure 8(a) illustrates a noticeable difference in absorbance spectra of uninfected and infected mosquitoes. Meanwhile, Figure 8(b) shows plot of partial least squares (PLS) models where more noticeable peaks at eight wavelengths (1000, 1413, 1515, 1711, 1801, 1893, 2109, 2246 nm) are observed. It must be noted that the spectrometer used in the study by Fernandes et al. (2018) consists of three sensors, namely a silicon sensor (350 nm–1000 nm) and two InGaAs sensors (1000–1800 nm, 1801–2500 nm). The authors explained that the two peaks in Figure 8(b) (1000 and 1800 nm) may either be due to molecular components of Zika virus or instrumental error due to transition between the sensors (Fernandes et al., 2018). Meanwhile, the spectral region between 1413 and 1515 nm is associated with O–H and N–H overtones, which might come from glycoproteins on viral envelopes or antimicrobial peptides from the mosquito immune system (Burns and Ciurczak, 2008; Dai et al., 2016; Xi et al., 2008). Information on other bands is limited during the study as NIR was just introduced in virology (Fernandes et al., 2018; Sakudo et al., 2006).

### 4 Raman spectroscopy

#### 4.1 Theory of Raman spectroscopy

**Figure 9:**
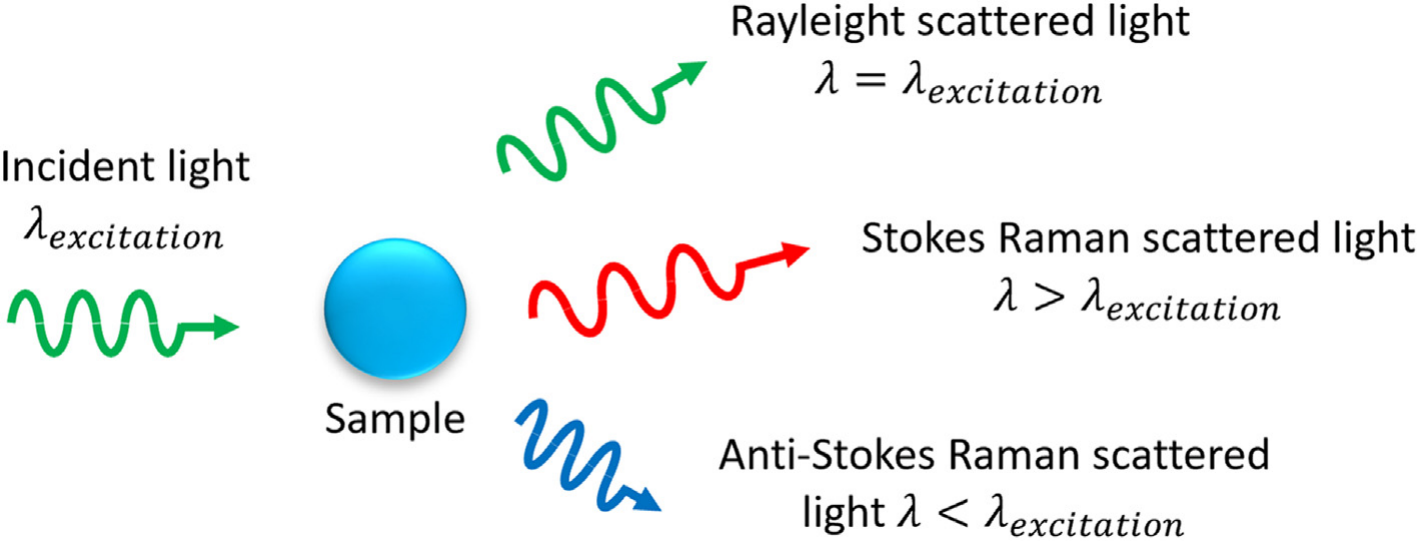
Effect of different types of scattering on the wavelength of scattered light.

Scattering occurs when the light of insufficient energy is incident on a molecule and is directed in different directions. When scattered light has the same frequency as the incident light, the scattering is considered elastic (Auner et al., 2018). While most light hits a molecule and is scattered elastically in the case of Rayleigh or Mie scattering, some light can be scattered at different frequencies, imparting energy to the molecule. Raman scattering is an example of inelastic scattering because the frequency of scattered light differs from that of incident light (Auner et al., 2018). Raman scattering can be divided into two types. Stokes Raman scattering occurs when the light has a longer wavelength upon scattering. In contrast, anti-Stokes Raman scattering occurs when the scattering light has a shorter wavelength than the incident light. Figure 9 illustrates the effects of scattering that occurs on the incident light.

Raman spectroscopy is a non-invasive method that employs Raman scattering. A laser with excitation wavelength of 532 nm is most commonly used in Raman spectroscopy (Bilal et al., 2017; Desai et al., 2020; Hermann et al., 2011). However, some studies used other wavelengths of laser such as 633 nm and 785 nm (Dou et al., 2020; Khan et al., 2018). It must be noted that different excitation wavelength causes a sample to exhibit different Raman shift. Furthermore, biomolecules such as DNA, RNA and protein in a virus have their own structural heterogeneity, thus, exhibiting different Raman spectra vibrations (Ruokola et al., 2014).

### 4.2 Techniques of Raman spectroscopy in past studies

Table 2 reveals the list of previous studies employing Raman spectroscopy. Spectra involving Raman spectroscopy is typically reported in terms of Raman shift, defined by the difference in wavenumber of the incident and scattered light.

**Table 2:** List of previous studies employing Raman spectroscopy on virus samples.

Author	Virus samples	Excitation wavelength (nm)	Raman shift (cm^−1^)	Findings
Hermann et al. (2011)	avipoxvirus (APV), adeno-associated virus (AAV)	532 nm	500 cm^−1^ to 3000 cm^−1^	• Several bands were visible in one of the spectra due to different contributions such as the interaction of the molecules with the Ag coating of the TERS tip. • Clear spectral variations between bands for each spectrum is observed in TERS spectra of AAV.
Bilal et al. (2017)	Dengue virus	532 nm	600–1800 cm^−1^	• The intense Raman bands at 1003, 1156 and 1516 cm^−1^ in healthy serum is suppressed when lactic acid solution is added. • The new peaks appeared around 750, 830, 925, 950, 1123, 1333, 1450, 1580 and 1730 cm^−1^.
Khan et al. (2018)	hepatitis B virus (HBV)	785 nm	500–1700 cm^−1^	• There is increase in Raman peaks (625, 678, 748, 810,820, 950, 1003, 1018 and 1275 cm^−1^) for infected samples. • There is a decrease in Raman peaks (128, 1220, 1250 and 1650 cm^−1^) for infected samples compared to healthy samples.
Dou et al. (2020)	MS2/HSV-2	633 nm	900–1700 cm^−1^	• The vibrational bands can be assigned to aliphatic group (1185–1195, 1340–1350, 1390 and 1479 cm^−1^), as well as amino and imino groups (1051–1068, 1120–1150 and 1168 cm^−1^). • There is less β-sheet in MS2 compared to α-helix/unordered protein secondary structure, reflected by smaller Raman peak at 1235 cm^−1^ and higher Raman peak at 1260 cm^−1^.
Desai et al. (2020)	RNA virus	532 nm	600–1800 cm^−1^	• Most of the bands in this range of Raman shift are caused by RNA moieties, particularly ribose sugars (900-1045 cm^−1^) and phosphate groups (1080-1100 cm^−1^). • Wilcoxon rank-sum test is used for each RNA specific constituent, such as the nitrogenous uracil base, ribose-phosphate, and A/G ring at 780, 1044, and 1480 cm^−1^, to determine whether these constituents reflect presence of RNA virus in samples.
Carlomagno et al. (2021)	SARS-CoV-2	785 nm	400 to 1600 cm^−1^	• Peaks located at 748, 922, 1048, 1126 and 1249 cm^−1^ differentiate salivary samples of healthy patients from that of infected and recovered patients. • Peaks at 1048 and 1126 cm^−1^, which are attributed to environment rich in aromatic amino acids, have significant difference between healthy samples and other samples.
Huang et al. (2021)	SARS-CoV-2	532 nm	400 to 1750 cm^−1^	• Based on amide I (1600–1690 cm^−1^) and amide III (1230–1300 cm^−1^) bands, spike protein in SARS-CoV-2 exhibits β-sheets/coils, in contrast to that of SARS-CoV and MERS-CoV exhibiting α-helix. • Raman spectra for spike protein in SARS-CoV-2 do not change before and after heat treatment, as primary and few secondary structures (responsible for Raman spectra) are not destroyed by heat treatment.

### 4.3 Evidence of Raman spectroscopy techniques

**Figure 10:**
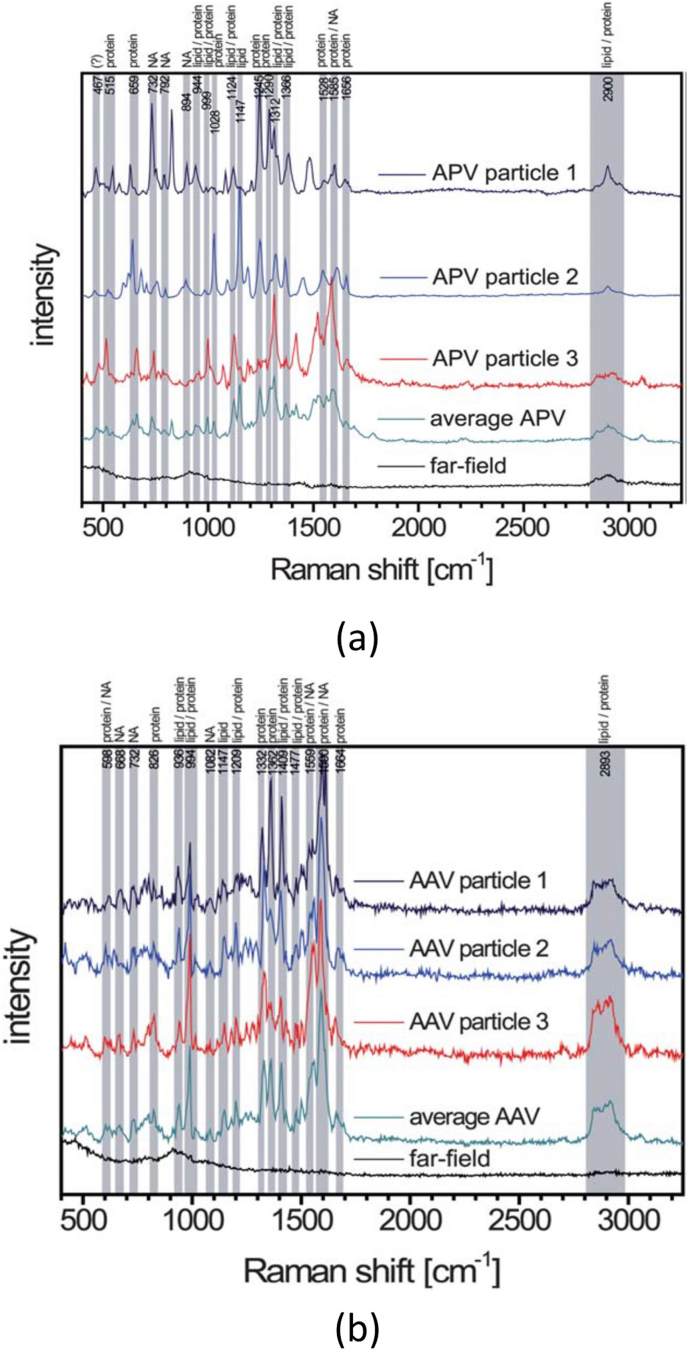
Raman spectra of (a) different particles of APV, and (b) different particles of AAV. Far-field is obtained from retracted tip (Hermann et al., 2011).

Hermann et al. (2011) studied avipoxvirus (APV) and adeno-associated virus (AAV) by conducting tip-enhanced Raman spectroscopy (TERS) with Raman shift ranging from 500 cm^−1^ to 3000 cm^−1^. The authors obtained Raman spectra of APV and AAV which are illustrated in Figure 10. A few bands such as in 1417 cm^−1^ and 1485 cm^−1^ in three single spectra for APV might be due to different contributions such as interactions of molecules with Ag coating of the TERS tip and gradient field effect (Ayars et al., 2000; Kneipp and Kneipp, 2013). On the other hand, several bands can be recognized and assigned to molecules such as amide I, amide III, and CH_2_ stretching (Hermann et al., 2011). As for AAV, clear spectral variations such as relative intensities of intensive bands between 1300 cm^−1^ and 1425 cm^−1^ and splitting of the band at about 1590 cm^−1^ are observed.

**Figure 11:**
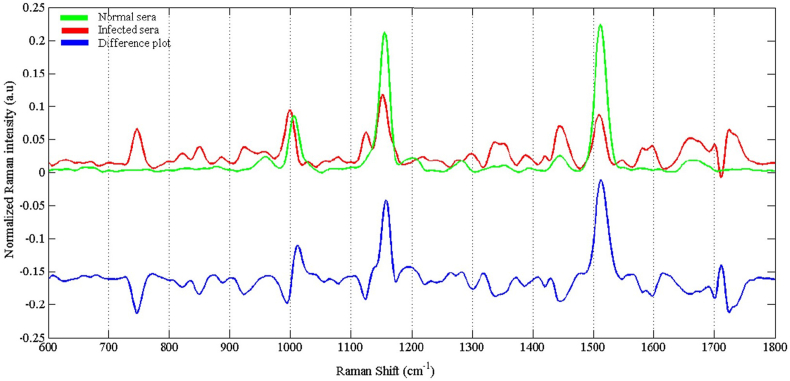
Raman spectra of normal sera (green) and dengue-infected sera (red). The difference in Raman intensity (blue) is also plotted on the same axis (Bilal et al., 2017).

Bilal et al. (2017) conducted Raman spectroscopy for screening dengue virus infection in human sera. Raman spectra of healthy and infected sera ranging from 600 cm^−1^ to 1800 cm^−1^ are illustrated in Figure 11. There are three Raman peaks with high intensity found in human sera, located at Raman shift of 1003 cm^−1^ (symmetric ring breathing mode of phenylalanine and β-carotene), 1156 and 1516 cm^−1^ (β-carotene) (Bilal et al., 2015; Movasaghi et al., 2007). These peaks are suppressed in infected sera, with new additional peaks (750, 830, 925, 950, 1123, 1333, 1450, 1580, 1680 and 1730 cm^−1^) formed at different Raman shifts, most probably due to high concentrations of lactate in the infected sera.

In another study conducted by Desai et al. (2020), preliminary result showed that most bands in Raman shift between 600 cm^−1^ and 1800 cm^−1^ are caused by RNA moieties, particularly in the ranges 900–1045 cm^−1^ (ribose sugars) and 1080–1100 cm^−1^ (phosphate groups). To find out whether these RNA moieties could determine the presence of RNA viruses, Wilcoxon rank-sum test is performed for RNA specific constituent, such as for nitrogenous uracil base (780 cm^−1^), ribose-phosphate (1044 cm^−1^) and A/G ring (1480 cm^−1^) (Chen et al., 2009; De Gelder et al., 2007). It is found that all of these peaks could differentiate healthy samples from infected samples with high statistical confidence (Desai et al., 2020).

**Figure 12:**
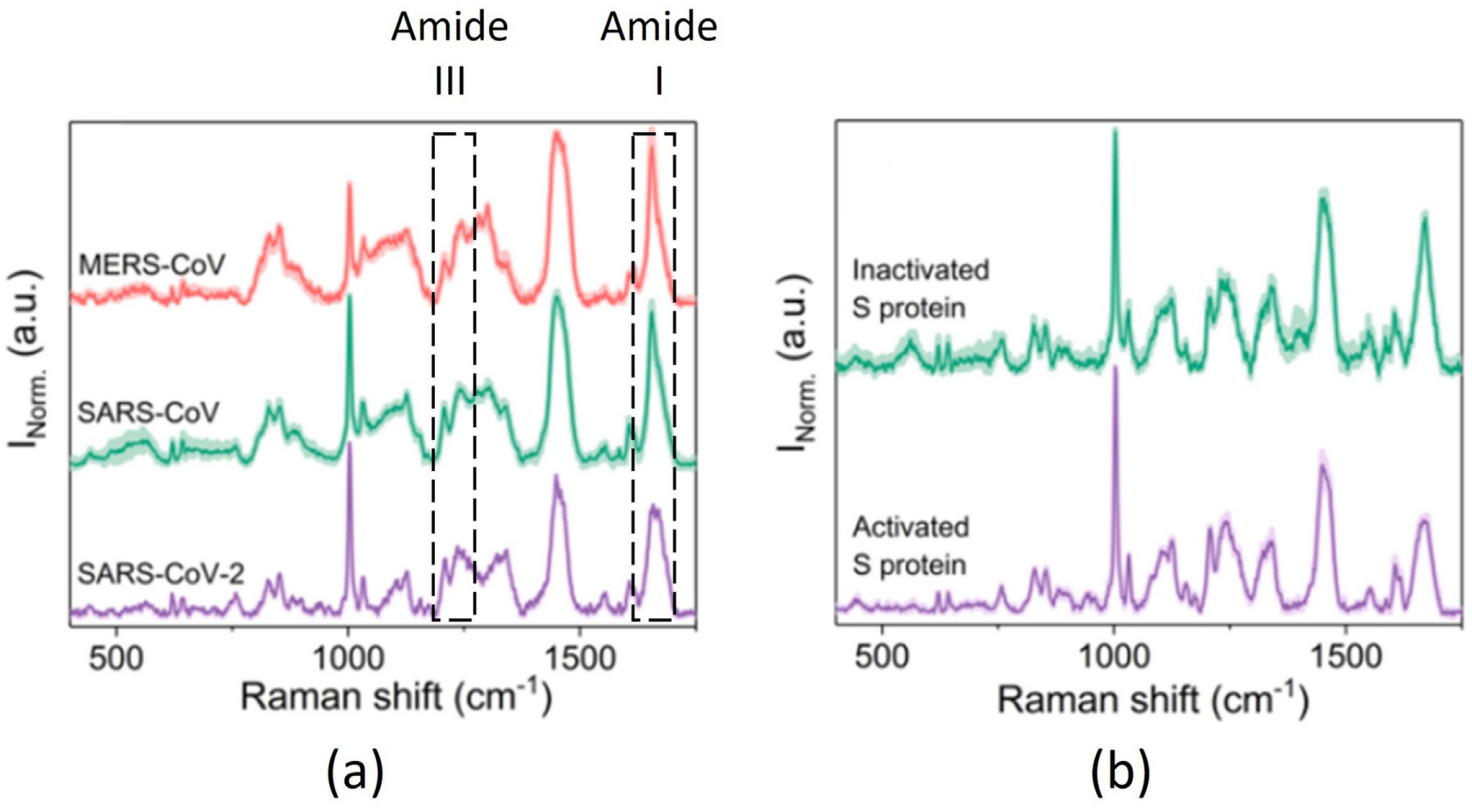
(a) Raman spectra of spike protein in different coronaviruses, and (b) Raman spectra of spike protein in SARS-CoV-2 before and after heat treatment (Huang et al., 2021).

Another study by Huang et al. (2021) on Raman spectra for spike protein of SARS-CoV-2 compared the spectra of activated spike protein of SARS-CoV-2 with other types of coronaviruses and inactivated spike protein of SARS-CoV-2. The characteristic Raman bands for amide I (1600–1690 cm^−1^) and amide III (1230–1300 cm^−1^) are highlighted in Figure 12(a). This reveals that Raman spectra for amide I/III bands in spike proteins of SARS-CoV-2 show more obvious β-sheets/coils, while spike proteins for SARS-CoV and MERS-CoV exhibits distinct α-helical components (Huang et al., 2021). The difference in amide I/III bands in SARS-CoV-2 compared to other coronaviruses contributes to specificity of Raman spectroscopy in detecting a particular virus. Furthermore, Figure 12(b) reveals that spike protein of SARS-CoV-2 shows an insignificant difference before and after heat treatment. This is because Raman spectra of spike protein in SARS-CoV-2 is attributed to vibration modes of primary structures and a few secondary structures, most of which are not destroyed by heat treatment (Tuma, 2005).

Previous Raman spectroscopy studies used a laser with an excitation wavelength of 532 nm (Bilal et al., 2017; Desai et al., 2020; Hermann et al., 2011; Huang et al., 2021). Previous studies that utilized laser with different excitation wavelength observed that peaks assigned to specific functional groups may be different (Dou et al., 2020; Khan et al., 2018; Wood et al., 2021). For example, amide III in α-helix protein secondary structure exhibits Raman peak of 1260 cm^−1^ with excitation wavelength of 633 nm, but different Raman peak (1275 cm^−1^) is assigned when excitation wavelength of 785 nm is used (Dou et al., 2020; Khan et al., 2018).

**Figure 13:**
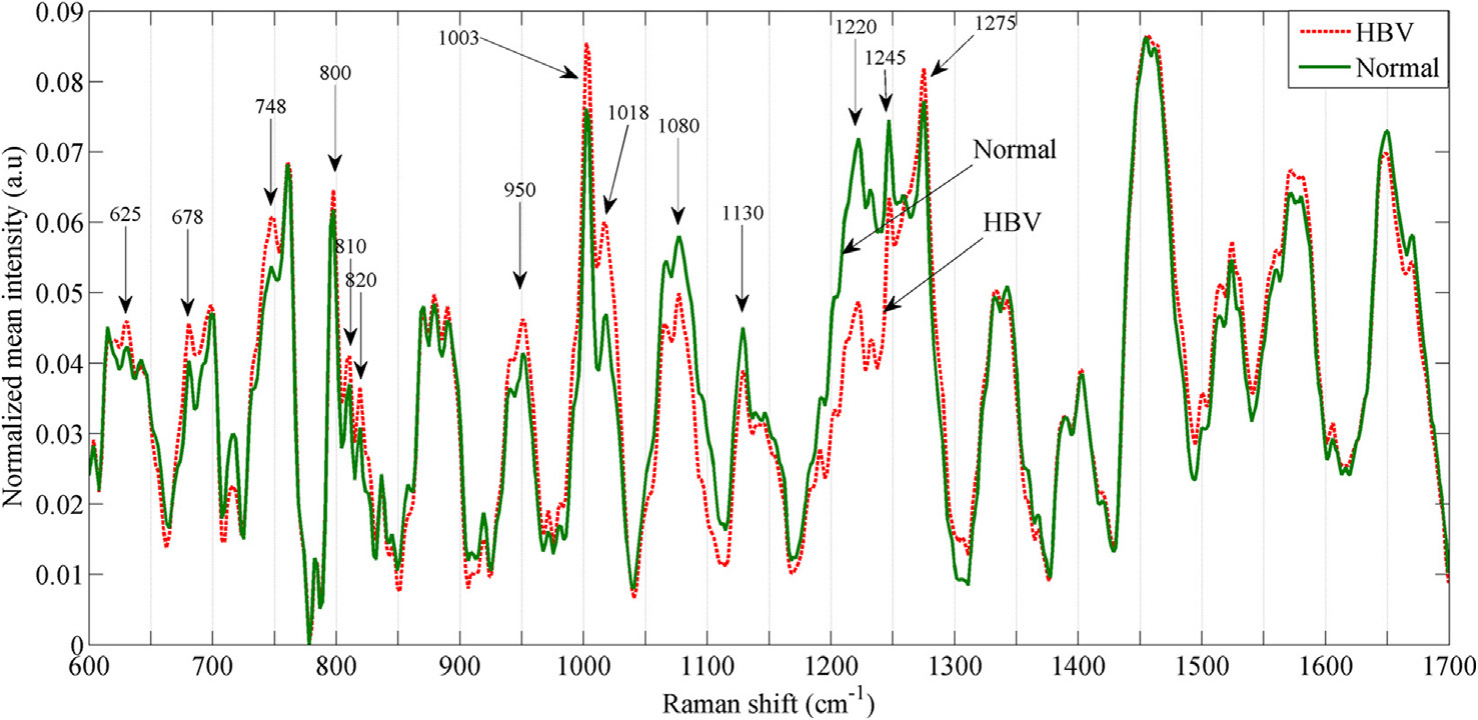
Raman spectra of healthy and HBV infected sera (Khan et al., 2018).

Another study by Khan et al. (2018) studied Raman spectra of HBV infected sera and healthy samples using laser with excitation wavelength of 785 nm. The Raman spectra of both samples are illustrated in Figure 13. It is observed that there is an increase (625, 678, 748, 810, 820, 950, 1003, 1018 and 1275 cm^−1^) and a decrease (128, 1220, 1250 and 1650 cm^−1^) in Raman peaks when the samples are infected with HBV. In the infected sera, increased glucose concentration, as indicated by higher Raman peak at 625 cm^−1^ (glucid) is mainly due to dysfunction of liver (Baker et al., 2016; Mustoi et al., 2011). Furthermore, there is an increase in albumin concentration in blood of infected person, as implied by increased Raman peaks of 1003 cm^−1^ with shoulder at 1018 cm^−1^ (phenylalanine) (Baker et al., 2016; Movasaghi et al., 2007; Rygula et al., 2013). The increased concentration of albumin is also implied by increased level of α-helix contents, assigned as increased Raman peak of 1275 cm^−1^ (amide III). The decreased Raman peaks of 1220 and 1250 cm^−1^ (coupling of C–N stretching and N–H bending) are assigned for amide III (β-sheet). This shows that β-sheet concentration is decreased in HBV infected samples, subsequently indicating that interferon secretion by lymphocytes occurs as immune response against HBV infection (De Gelder et al., 2007).

In a study by Dou et al. (2020), Raman spectroscopy with excitation wavelength of 633 nm is also performed on MS2 bacteriophage, apart from AFM-IR. Raman spectra of different MS2 particles exhibits different pattern from MIR absorbance spectra of MS2. The vibrational bands can be assigned to aliphatic group (1185–1195, 1340–1350, 1390 and 1479 cm^−1^), as well as amino and imino groups (1051–1068, 1120–1150 and 1168 cm^−1^) (Deckert-Gaudig et al., 2012, 2016). The position of amide III bands located at 1235–1260 cm^−1^ can be used to determine secondary structure of protein as β-sheet at amide III and α-helix/unordered protein secondary structure exhibit Raman peaks of 1235 cm^−1^ and 1260 cm^−1^ respectively (Deckert-Gaudig et al., 2016; Kurouski et al., 2015). Smaller Raman peak at 1235 cm^−1^ reflects less β-sheet in MS2 compared to α-helix/unordered protein secondary structure.

**Figure 14:**
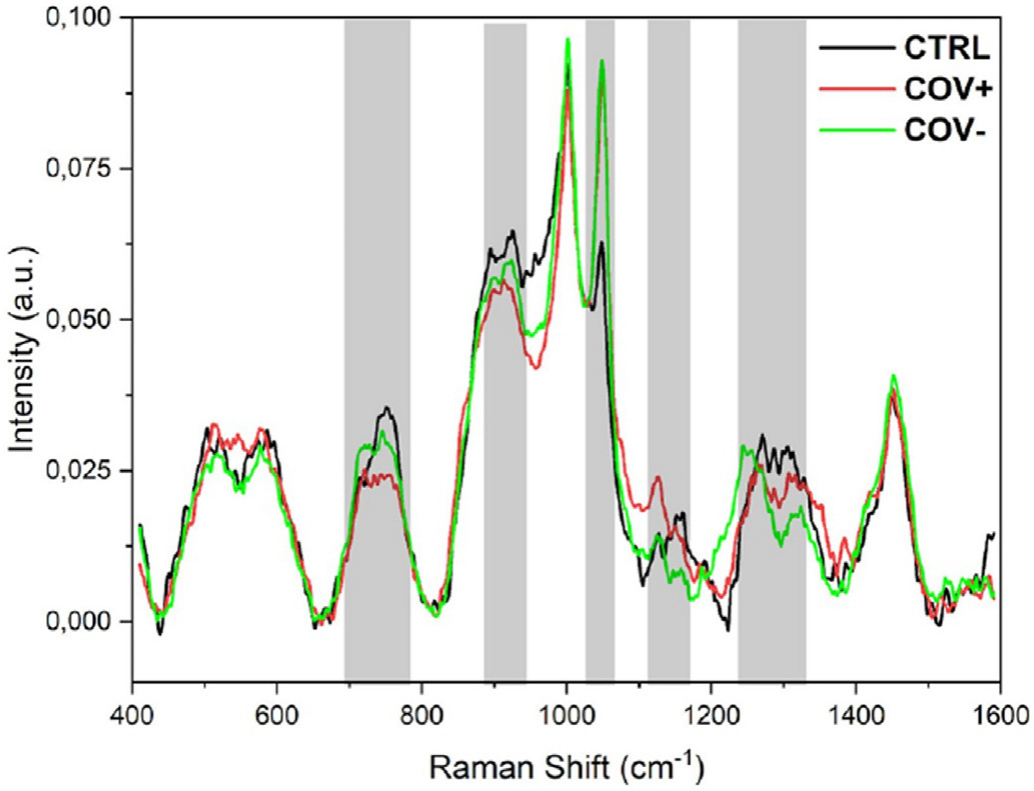
Raman spectra of salivary sample from healthy patients (CTRL), COVID-19 infected patients (COV+), and patients recovered from COVID-19 (COV-). The difference in spectra is highlighted by dark bands (Carlomagno et al., 2021).

A study carried out by Carlomagno et al. (2021) discriminates salivary sample from patients infected with COVID-19 (COV+), patients recovered from COVID-19 (COV-) and healthy patients (CTRL). The study was carried out using laser with an excitation wavelength of 785 nm. Figure 14 reveals that peaks located at 748, 922, 1048, 1126 and 1249 cm^−1^, as highlighted by the dark bands, differentiate Raman spectra of different groups. Peaks at 1048 cm^−1^ and 1126 cm^−1^ are the most significant in differentiating Raman spectra of the healthy patient from those of infected and recovered patients respectively. The significant difference at 1048 cm^−1^ and 1126 cm^−1^ are associated with an environment rich in aromatic amino acids, particularly tryptophan and phenylalanine. The presence of aromatic amino acids in virus spike glycoproteins caused regions rich in tryptophan, which are involved in the interaction between SARS-CoV-2 and Angiotensin Converting Enzyme type 2 (ACE2) (Liao et al., 2015).

## 5 Fluorescence spectroscopy

### 5.1 Theory of fluorescence spectroscopy

Fluorescence is the phenomenon where fluorophore in the sample absorbs light at a shorter wavelength and emits light at a longer wavelength, usually in visible spectrum. Fluorophore usually contains aromatic rings such as fluorescein, tyrosine and tryptophan (Bose et al., 2018).

**Figure 15:**
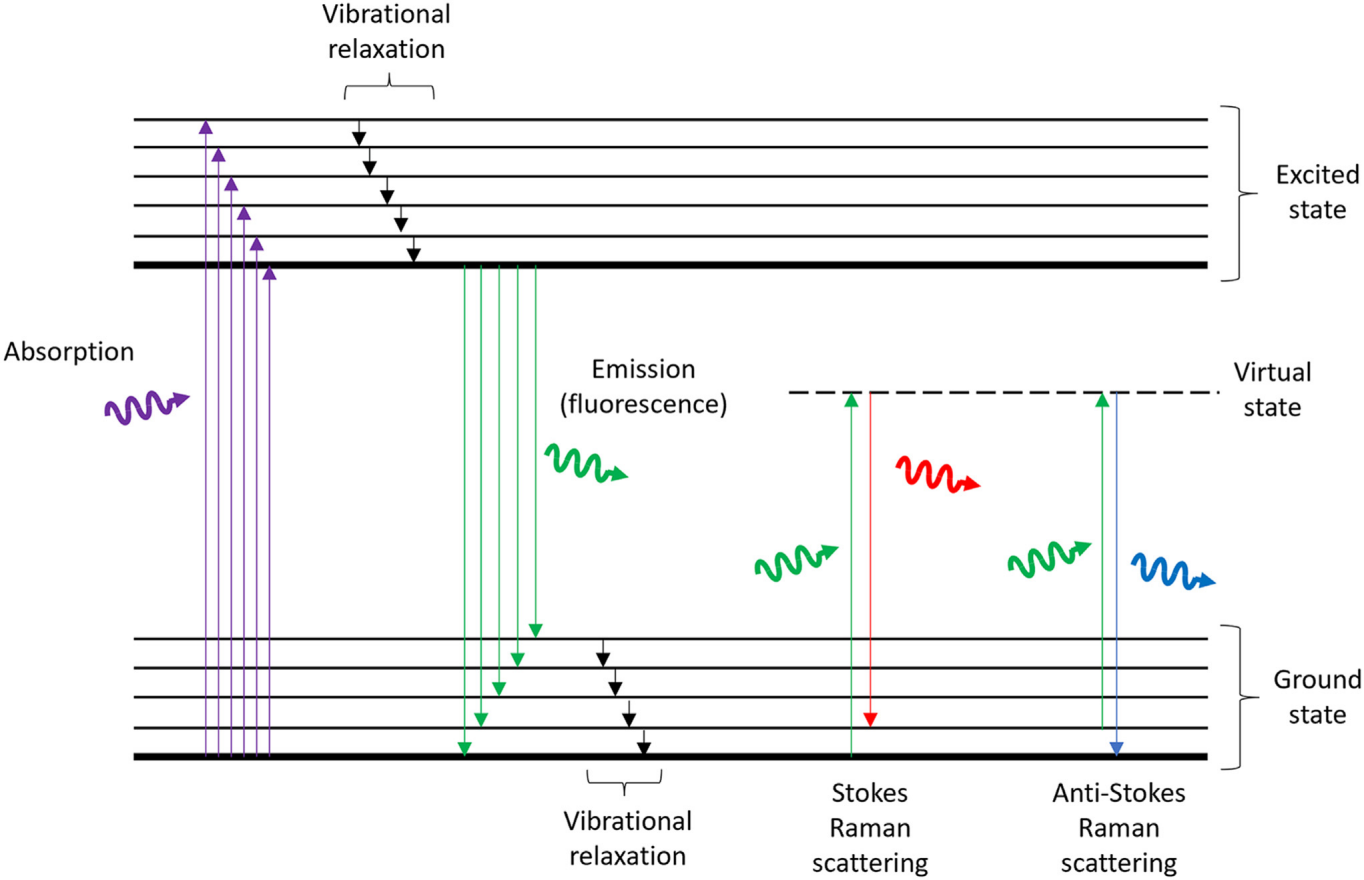
Electronic transition and vibrational relaxation in fluorescence and Raman scattering.

Fluorescence occurs when an electron transitions from singlet ground state to singlet excited state during light absorption, and jumps to an allowable vibrational level before returning to ground state (Bose et al., 2018). These processes are illustrated in Figure 15. Both fluorescence and Raman scattering involve absorption and emission of light at different wavelengths. However, fluorescence occurs when the electron jumps from the ground state to the excited state, while Raman scattering involves the electron transitioning between the ground state and virtual state (Auner et al., 2018; Bose et al., 2018). Fluorescence spectroscopy on the detection of virus is usually supplemented with fluorescent tags such as aptamers, nanoparticles, and quantum dots to aid fluorescence quenching or improve fluorescence signal from viral particles (Lukose et al., 2021; Pramanik et al., 2021; Yang et al., 2020).

### 5.2 Techniques of fluorescence spectroscopy in past studies

Table 3 reveals the list of previous studies employing fluorescence spectroscopy in detecting virus in samples. The description includes excitation wavelength of laser used in the study and other relevant information related to molecules used in addition to virus samples.

**Table 3:** List of previous studies employing fluorescence spectroscopy on virus samples.

Author	Virus samples	Description	Findings
Huang et al. (2016)	Hepatitis B e antigen (HBeAg)	• Laser with excitation wavelength of 485 nm is used. Emission wavelength is fixed at 528 nm • Aptamer linked with gold nanoparticle is used as aptasensor in detecting HBeAg. The reaction time is 2 min	• Fluorescence intensity of aptasensor at 528 nm increases at higher concentration of HBeAg • The increase does not occur when other proteins such as HBcAg and trypsin are tested
Yang et al. (2020)	Japanese encephalitis virus (JEV)	• Laser with excitation wavelength of 290 nm is used • Molecularly imprinted polymer (MIP) acts as fluorophore in detecting JEV • The reaction time for fluorescence is 20 min	• JEV enhanced peak of fluorescence intensity of MIP at 340 nm. • The peak of fluorescence intensity of MIP is higher with increasing concentration of JEV. • Significantly higher fluorescence intensity of MIP is observed in presence of JEV, compared to other viruses.
Piva et al. (2020)	Human respiratory syncytial virus (hRSV)	• Laser with excitation wavelength of 290 nm is used • Interaction between M_2-1_ and flavonoids (HST and HSD) are studied based on fluorescence specta	• Both flavonoids effectively quenches fluorescence signal from M_2-1_ protein. • HST has higher binding affinity towards M_2-1_ protein compared to HSD, indicated by lower fluorescence intensity for HST at similar concentrations.
Santos et al. (2020)	Chikungunya virus (CHIKV), dengue virus (DENV)	• Laser with excitation wavelength ranging from 250 – 320 nm is used, with steps of 10 nm • Additional model such as PARAFAC and PLSDA are employed	• Peak fluorescence intensity between 300 – 415 nm is due to fluorophore. Infected samples (CHIKV and DENV) exhibit lower peaks at this range. • PARAFAC-QDA is proved to be the most effective model in differentiating all three types of samples (uninfected, CHIKV, DENV)
Pramanik et al. (2021)	SARS-CoV-2	• Rh-6G dye conjugated DNA aptamer-attached gold nanostar is used to detect SARS-CoV-2 antigen via fluorescence • Laser with unspecified excitation wavelength is used to cause fluorescence on the dye. The reaction time is set as 10 min	• In the absence of SARS-CoV-2 antigen or virus particles, gold nanostar effectively quenches the fluorescence signal from the dye. • SARS-CoV-2 spike protein binds with DNA aptamer, increasing the distance between gold nanostar and the dye. Consequently, the fluorescence signal increases. This does not occur when spike protein of flu virus or rotavirus is used.

### 5.3 Evidence of fluorescence spectroscopy techniques

A study by Huang et al. (2016) developed aptasensor, consisting of aptamer linked gold nanoparticles in detecting hepatitis B e antigen (HBeAg) in a sample. The study used laser with excitation wavelength of 485 nm and detected fluorescence from sample at emission wavelength of 528 nm. The aptasensor is proven to detect HBeAg with high sensitivity and specificity. The result showed that fluorescence intensity at 528 nm increases with higher concentration of HBeAg. This is because the aptasensor labelled with fluorescent dye hybridizes with Dabcyl modified probe Q to form DNA duplex in the absence of HBeAg (Huang et al., 2016). This leads to quenching of fluorescence signal from the aptasensor, which explains low fluorescence intensity in the absence of HBeAg. The increase in fluorescence intensity does not occur when other proteins such as HBcAg and trypsin are tested at higher concentration, indicating high specificity of the aptasensor.

**Figure 16:**
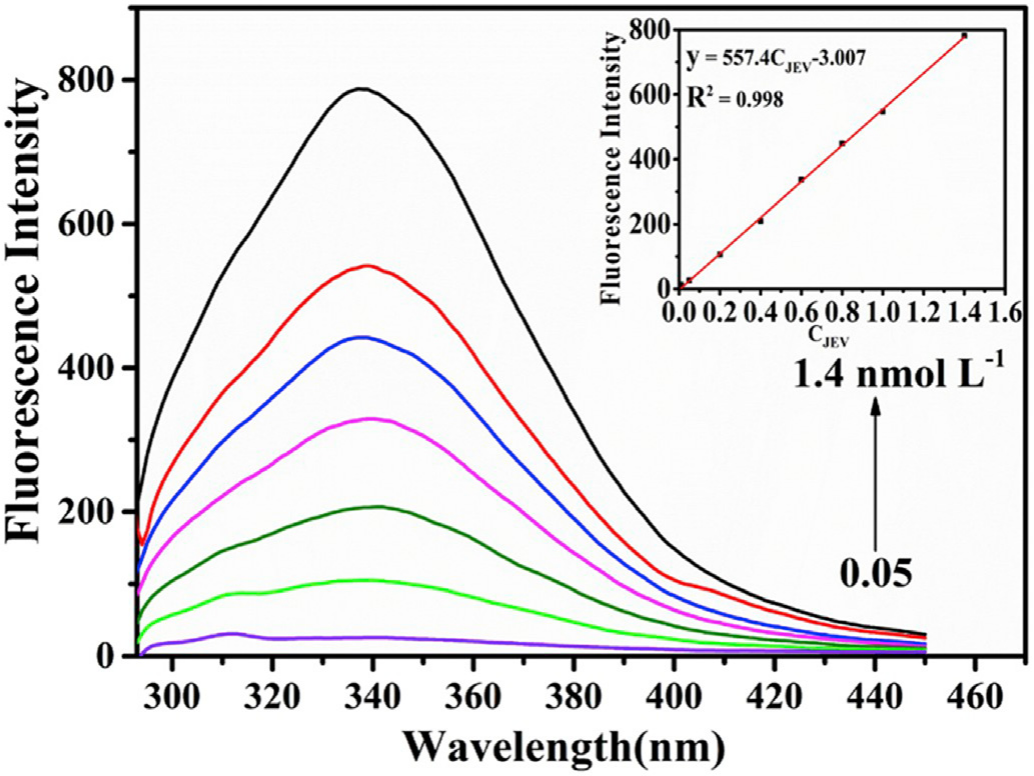
Fluorescence spectra of MIP at different concentrations of JEV (Yang et al., 2020).

Yang et al. (2020) studied on detection of Japanese encephalitis virus (JEV) by synthesizing viral molecularly imprinted polymers (MIP) based on metal organic framework. The study used a laser with an excitation wavelength of 290 nm and the reaction time is 20 min. The study showed that the presence of JEV improved the fluorescence signal from MIP due to energy transferred from JEV to MIP (Yang et al., 2020). Figure 16 showed that the fluorescence intensity peak of MIP at 340 nm is higher with increasing concentration of JEV, which enhances sensitivity in detecting JEV. It is also found that MIP has high specificity in detecting JEV, as its fluorescence intensity is significantly higher in the presence of JEV, compared to other viruses such as hepatitis A, rabies and leprosy.

**Figure 17:**
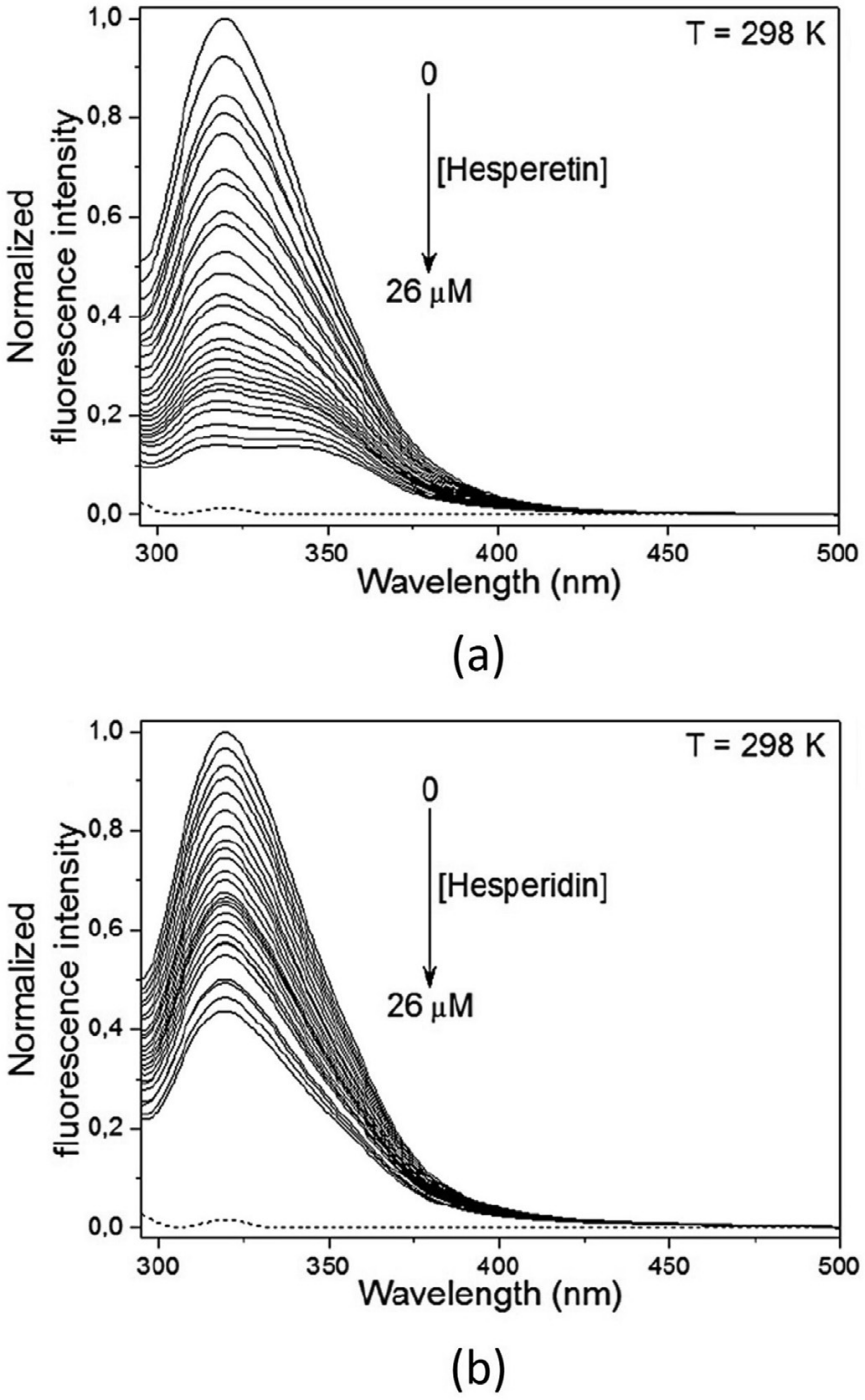
Fluorescence spectra of hRSV MS_2-1_ protein in absence and presence of (a) hesperetin (HST), and (b) hesperidin (HSD) at different concentrations (Piva et al., 2020).

A study conducted by Piva et al. (2020) utilized fluorescence in studying the interaction between M_2-1_ protein in human respiratory syncytial virus (hRSV) with hesperetin (HST) and hesperidin (HSD) as flavonoids. The study used laser with excitation wavelength of 290 nm. Figure 17(a) and 17(b) show that peak fluorescence intensity due to M_2-1_ protein decreases with higher concentration of respective flavonoids. This is because both flavonoids affect molecular nanoenvironment of fluorophores in the protein, effectively quenching the fluorescence intensity (Piva et al., 2020). In addition, lower fluorescence intensity is observed when HST at concentration of 26 μM is present in the sample, compared to HSD at similar concentrations. This indicates that HST is more effective in quenching fluorescence from M_2-1_ protein. This is because glycosylation in HST decreases the binding affinity of protein, consequently reducing the interaction of HST with M_2-1_ protein (Cao et al., 2009; Piva et al., 2020).

Santos et al. (2020) conducted a study on differentiating healthy serum samples from infected serum samples caused by chikungunya virus (CHIKV) and dengue virus (DENV). The laser used in the study has excitation wavelength ranging from 250 nm to 320 nm, with 10 nm increment. Fluorescence spectra for both uninfected and infected serum samples revealed that the high peak is present at emission wavelength, ranging from 300 nm to 415 nm. This occurs due to endogenous fluorophores in the samples (Conklin et al., 2009). However, the infected samples exhibit lower peak, which may be due to chemical virus-cell interactions (Santos et al., 2020). The study further employed several models to differentiate all three types of samples, and concluded that parallel factor analysis with quadratic discriminant analysis (PARAFAC-QDA) is the most efficient model.

**Figure 18:**
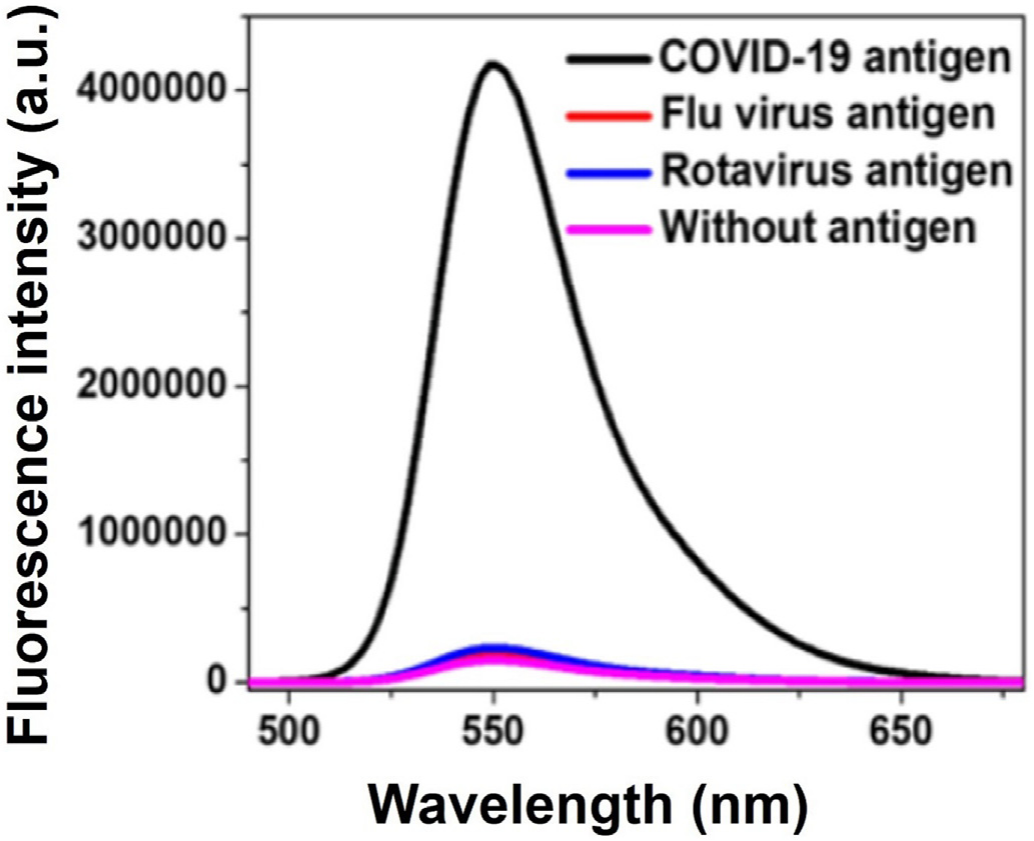
Fluorescence spectra of Rh-6G conjugated DNA aptamer-attached GNS in absence and presence of SARS-CoV-2 antigen (10 pg/mL), flu virus (100 pg/mL) and rotavirus (100 pg/mL) (Pramanik et al., 2021).

In a recent study conducted by Pramanik et al. (2021), fluorescence is used to detect SARS-CoV-2 antigen or virus particles. Gold nanostar (GNS) is attached with Rhodamine 6G (Rh-6G) dye conjugated DNA aptamer. This is because GNS can effectively quench fluorescence signal from the dye via nanoparticle surface energy transfer (NSET) (Carnevale et al., 2018). Based on Figure 18, the aptamer-attached GNS exhibits high peak fluorescence intensity at around 550 nm when SARS-CoV-2 antigen is present. This is because the aptamer binds with spike protein of SARS-CoV-2, increasing the distance between GNS and the dye. Consequently, the GNS cannot quench fluorescence signal from the dye, and high peak fluorescence intensity is observed (Pramanik et al., 2021). GNS is shown to be specific with SARS-CoV-2 antigen or virus particles as high peak fluorescence intensity is observed when SARS-CoV-2 antigen at concentration of 10 pg/mL is present. This is not observed for other antigens such as flu virus or rotavirus, both of which are present at concentrations of 100 pg/mL respectively.

## 6 Conclusion

It has been shown that UV spectroscopy research is not very much conducted in recent previous studies involving virus infected samples. At sufficiently high intensities of UV, RNA of the virus may degrade via dimer formation mechanism as shown in Figure 1. In fact, UV spectroscopy of virus samples have been related to studies involving irradiation of the virus. Meanwhile, several studies have focused on virus samples that employ IR and Raman spectroscopy to determine the molecules present via vibration and overtones of the molecules. Out of all three types of IR radiation, NIR and MIR are most commonly used in studies involving virus samples. MIR’s spectral data can be easily processed because data acquisition involving MIR has been equipped with either Fourier transform or atomic force microscopy. NIR usually requires simpler instrumentation than MIR, but spectral data acquired from NIR does not directly provide peaks that indicate vibrational modes of certain molecule. Thus, analysis of NIR spectral data usually involves additional step, which includes PCA and PLS plot. Raman spectroscopy is also commonly used in the study involving virus samples. Instrumentation involving Raman spectroscopy is more complicated, but the spectral data obtained is very easy to analyse and does not usually require additional steps. Raman peaks from spectral data can be directly analysed for the determination of molecules via their own unique vibrational modes. Fluorescence spectroscopy has been proven to detect certain virus with high specificity. Unlike IR and Raman spectroscopy which relies on assignment of peaks in spectra, fluorescence spectroscopy relies on the interaction between viral particles and fluorescent tags either for improvement or quenching of fluorescence signal from virus.

Due to destructive properties of UV, future studies may not use UV spectroscopy to study virus samples. However, it is possible to still conduct UV spectroscopy on virus, provided that the intensity of UV is not sufficiently high to cause degradation of virus samples. In contrast, IR and Raman spectroscopy will be continuously used for future studies, especially with the recently emerged COVID-19 pandemic. Both spectroscopy methods are non-invasive and provide useful information about the virus molecules. Meanwhile, fluorescence spectroscopy methods are also expected to be used continuously for future studies as they have been proven to detect viral particles with high specificity, despite additional fluorescent tags and required reaction time.

## Declarations

### Author contribution statement

All authors listed have significantly contributed to the development and the writing of this article.

### Funding statement

This work was supported by Universiti Malaysia Sabah through grant number SDK0163-2020.

### Data availability statement

Data included in article/supp. material/referenced in article.

### Declaration of interest’s statement

The authors declare no conflict of interest.

### Additional information

No additional information is available for this paper.
